# ZOOMICS: Comparative Metabolomics of Red Blood Cells From Guinea Pigs, Humans, and Non-human Primates During Refrigerated Storage for Up to 42 Days

**DOI:** 10.3389/fphys.2022.845347

**Published:** 2022-03-21

**Authors:** Lorenzo Bertolone, Hye Kyung H. Shin, Jin Hyen Baek, Yamei Gao, Steven L. Spitalnik, Paul W. Buehler, Angelo D’Alessandro

**Affiliations:** ^1^Department of Biochemistry and Molecular Genetics, University of Colorado Denver – Anschutz Medical Campus, Aurora, CO, United States; ^2^Center for Biologics Evaluation and Research, Food and Drug Administration, Silver Spring, MD, United States; ^3^Department of Pathology and Cell Biology, Columbia University, New York, NY, United States; ^4^Department of Pathology, University of Maryland School of Medicine, Baltimore, MD, United States; ^5^Department of Pediatrics, Center for Blood Oxygen Transport and Hemostasis, University of Maryland School of Medicine, Baltimore, MD, United States; ^6^Department of Medicine, Division of Hematology, University of Colorado Denver – Anschutz Medical Campus, Aurora, CO, United States

**Keywords:** comparative biology, erythrocyte, rodent, hemolysis, metabolomics, ascorbate

## Abstract

Unlike other rodents, guinea pigs (*Cavia porcellus*) have evolutionarily lost their capacity to synthesize vitamin C (ascorbate) *de novo* and, like several non-human primates and humans, rely on dietary intake and glutathione-dependent recycling to cope with oxidant stress. This is particularly relevant in red blood cell physiology, and especially when modeling blood storage, which exacerbates erythrocyte oxidant stress. Herein we provide a comprehensive metabolomics analysis of fresh and stored guinea pig red blood cell concentrates (*n* = 20), with weekly sampling from storage day 0 through 42. Results were compared to previously published ZOOMICS studies on red blood cells from three additional species with genetic loss of L-gulonolactone oxidase function, including humans (*n* = 21), olive baboons (*n* = 20), and rhesus macaques (*n* = 20). While metabolic trends were comparable across all species, guinea pig red blood cells demonstrated accelerated alterations of the metabolic markers of the storage lesion that are consistent with oxidative stress. Compared to the other species, guinea pig red blood cells showed aberrant glycolysis, pentose phosphate pathway end product metabolites, purine breakdown products, methylation, glutaminolysis, and markers of membrane lipid remodeling. Consistently, guinea pig red blood cells demonstrated higher end storage hemolysis, and scanning electron microscopy confirmed a higher degree of morphological alterations of their red blood cells, as compared to the other species. Despite a genetic inability to produce ascorbate that is common to the species evaluated, guinea pig red blood cells demonstrate accelerated oxidant stress under standard storage conditions. These data may offer relevant insights into the basal and cold storage metabolism of red blood cells from species that cannot synthesize endogenous ascorbate.

## Introduction

While originally a wild food source for humans, guinea pigs (*Cavia porcellus*) were domesticated ∼5,000 years ago, before dispersing through pre-Columbian exchange networks in Peru, Bolivia, Colombia, the Caribbean, Belgium, and the United States (Lord et al., 2020). An accidental discovery in 1907 showed that scurvy could be induced in guinea pigs, and a later discovery revealed impaired vitamin C (i.e., ascorbate) synthesis in these rodents, owing to loss-of-function mutations in L-gulonolactone oxidase (GULO) genes, which halts conversion of gulonolactone to ascorbate (Burns, 1957). Among mammals, loss of ascorbate synthesis capacity is only observed in anthropoid primates (including humans), some bats, and guinea pigs (Chatterjee, 1973). In pre-clinical research, guinea pigs are relevant models of ascorbate deficiency disorders (May, 2011; Søgaard et al., 2014). Guinea pig models are also valuable for studying coagulation cascades (André et al., 1996), developmental and reproductive toxicology (Rocca and Wehner, 2009), infectious diseases (Padilla-Carlin et al., 2008; Hensel and Arenas-Gamboa, 2018; Ghasemi et al., 2021), and organophosphate toxicity (Lambré et al., 1982; Atchison et al., 2004; Ovsyannikova et al., 2014).

Within the context of RBC transfusion, rodent (Baek et al., 2012; Howie et al., 2019; Williams et al., 2019), canine (Klein, 2017), and porcine (Clendenen et al., 2017) transfusion studies provide orthogonal insights into the diverse physiology of RBCs prior to and after refrigerator storage. Because blood banking, combined with subsequent RBC transfusions. is critical for millions of patients every year, basic science advances in blood quality assessment and storage quality improvement remain relevant. Although meaningful information can be obtained from non-clinical studies, there is no standard accepted pre-clinical model for human blood transfusion.

Understanding common biological features that impact RBC physiology across species may offer insight into improving blood preservation. Here we consider mammalian species that rely on dietary intake of ascorbate. However, guinea pigs and primates demonstrate RBC features that may not be expected. For example, healthy guinea pigs have hematocrits of 35–47% (Genzer et al., 2019), comparable to the 39–45% for macaques, 33–46% for baboons (Valeri and Ragno, 2006), and 35–49% for humans. Hemoglobin ranges for healthy guinea pigs are 13 ± 0.9 g/dL (Genzer et al., 2019), with corresponding levels of 12.5 ± 1.1 g/dL for macaques and 12.6 ± 1.2 g/dL for baboons (Harewood et al., 1999), respectively (Chen et al., 2009), and 12.0–17.5 g/dL for humans. In addition, RBC distribution widths are 13.0 ± 1.6 for guinea pigs (Spittler et al., 2021), 13.0 ± 0.7% and 12.9 ± 1.0 for macaques and baboons (Mahaney et al., 2005), respectively, and similar values for humans (Chen et al., 2009). Finally, guinea pig, macaque, baboon, and human RBC disk diameters average 7.1, 7.4, 7.8, and 7.9 microns, respectively, when measured on dry films (Gregory, 2000). Despite these similar parameters, guinea pig RBCs—like those of most rodents—are shorter lived than primate RBCs (Siegel and Walton, 2020). Indeed, guinea pig RBCs have an average life span of 81 days range 77–91 days (Edmondson and Wyburn, 1963), which is shorter than that of macaques (98 ± 21 days) (Fonseca et al., 2016, 2018), baboons (∼100 days) (Valeri et al., 1981), or humans (100–120 days), but longer than that of mice [55–60 days (Kaestner and Minetti, 2017)].

Alterations of energy and redox metabolism are a hallmark of RBC aging *in vivo* (D’Alessandro et al., 2013; Jamshidi et al., 2020; Mykhailova et al., 2020) and *in vitro* [i.e., during refrigerated storage in blood banks (Yoshida et al., 2019)]. In particular, given the primary role of oxidant stress in the progression of the so-called “RBC storage lesion” (Yoshida et al., 2019), it has been posited that that the ascorbate pathway is diminished, but modifiable with supplementation of ascorbate in stored RBCs, thereby providing a storage lesion mitigation strategy (Stowell et al., 2013; Pallotta et al., 2014; Sanford et al., 2017). However, although the trends are well established in human (D’Alessandro et al., 2019, 2021a), non-human primate (Bertolone et al., 2020; Stefanoni et al., 2020), or, even, rodent RBCs [e.g., mice (Zimring et al., 2014; Howie et al., 2019), rats (Williams et al., 2019)], little is known about the impact of refrigerated storage on guinea pig RBC metabolism, which we hypothesize could be impaired as a result of their genetic loss of ascorbate synthesis.

As part of our Zoomics project, we are determining the specie-specific RBC metabolic processes that may affect blood storage quality, which is directly relevant to animal species specific transfusion in veterinary medicine, defining and understanding relevant surrogate models of human transfusion. To this end, previous studies identified parallel and divergent metabolomics adaptation in fresh and stored RBCs in three primate species: humans, rhesus macaques, and baboons (Bertolone et al., 2020; Stefanoni et al., 2020). These results highlighted unique adaptation in arginine metabolism across primates, which could be relevant to arginine-derived RBC synthesis of nitric oxide, critical to vascular responsiveness to hypoxia (Doctor and Stamler, 2011) and transfusion efficacy (Bennett-Guerrero et al., 2007; Kanias et al., 2013; Reynolds et al., 2018). In the present study, metabolomics analyses were performed on freshly collected and stored guinea pig RBCs, with weekly sampling until the end of the storage period (i.e., 42 days, to facilitate direct comparison to humans). Thus, the results overlapped with identical blood storage studies we performed on RBCs from primates (humans, rhesus macaques, and baboons) (Bertolone et al., 2020; Stefanoni et al., 2020). Despite the lack of *de novo* ascorbate synthesis in all of the species in the current study, metabolic markers of guinea pig RBCs demonstrated more rapid and severe presentation of the storage lesion compared to primates. Therefore, poor post-transfusion recovery and increased hemolysis (D’Alessandro et al., 2020) would be expected following human equivalent storage of guinea pig RBCs. This expectation is based on our understanding of aberrations in the identified metabolites (Paglia et al., 2016), and as a function of lipid remodeling (Thomas et al., 2021) and altered RBC morphology (D’Alessandro et al., 2012; Roussel et al., 2021). Interestingly, this post-transfusion outcome is a genetically-regulated trait of human RBCs (Van ‘T Erve et al., 2015; D’Alessandro et al., 2021a).

## Materials and Methods

### Ethical Statement

All experimental protocols were approved by the relevant institutional committees. Specifically, animal studies were performed according to FDA White Oak Animal Care and Use protocols 2009-25 (for guinea pigs) and 2018-31 (macaques and baboons). Human blood was collected under informed consent according to NIH study IRB #99-CC-0168 “Collection and Distribution of Blood Components from Healthy Donors for *in vitro* Research Use” under an NIH-FDA material transfer agreement and in compliance with the Declaration of Helsinki.

### Blood Collection

Whole blood was collected in syringes into acid citrate dextrose solution A (Becton Dickenson, Franklin Lakes, NJ, United States) to make up 15% of the volume. Guinea pig blood was collected into syringes using sterile PE10 surgically implanted carotid artery catheter coupled to 23 G needle and 4-way stopcock ketamine/xylazine (40, 5 mg/kg) anesthetized 2.5-month-old Hartley guinea pigs (*n* = 20, 10 male, 10 female) according to FDA White Oak animal care and use protocol 2009-25. All blood donor guinea pigs originated from Charles Rivers Laboratories. Non-human primate blood was collected into a syringe using a 20 G needle from the femoral vein of 5-year-old rhesus macaques (*Macaca mulatta*, *n* = 20; 10 male/10 female) and olive baboons (*Papio anubis*—*n* = 20; 10 male/10 female) under ketamine/dexmedotomidine (7 mg/kg/0.2 mg/kg, intramuscular) anesthesia according to FDA White Oak animal care and use protocol 2018-31. All blood donor rhesus macaques originated from the same colony located at Morgan Island, South Carolina and all blood donor olive baboons originated from Southwest National Primate Research Center, San Antonio Texas. Human blood was collected into a syringe using a 16 G needle from the median cubital vein of 30–75-year-old human volunteers (*n* = 21; 11 male/10 female) under informed consent according to NIH study IRB #99-CC-0168 “Collection and Distribution of Blood Components from Healthy Donors for *in vitro* Research Use” under an NIH-FDA material transfer agreement.

### Blood Processing and Storage

Guinea pig, rhesus macaque, olive baboon and human blood was processed, stored, and sampled the same way. Collected blood was individually processed by passage through a pediatric leukoreduction filter (Haemonetics, Braintree, MA, United States). Approximately, 20–30 ml of leukoreduced whole blood was then centrifuged at 2,000 rpm for 10 min, plasma was removed, and 0.45 ml of AS-3 (Haemonetics, Braintree, MA, United States) was added for every 1 ml of packed RBCs. The RBCs in AS-3 preservative solution (total volume ∼12–15 ml) were transferred to a sterile customized single port bag through a sterile self-sealing sampling site coupler port (Fenwal, Lake Zurich, IL, United States). The volume modified storage bags (Hemanext Inc., Lexington, MA, United States), were designed to hold 20 ml volumes and approximate the plasticizer composition of standard units that incorporate polyvinylchloride (PVC) and phthalate plasticizers (DEHP and MEHP). Processing procedures were performed in a biosafety cabinet under aseptic conditions the morning of the blood collections and red blood cells were maintained under standard refrigerator storage (4–6°C). RBCs and supernatants were separated *via* centrifugation (2,500 rpm) upon sterile sampling of each unit on days 0, 7, 14, 21, 28, 35, and 42.

### Ultra-High-Pressure Liquid Chromatography-Mass Spectrometry Metabolomics and Lipidomics

A volume of 50 μL of frozen RBC aliquots was extracted 1:10 in ice cold extraction solution (methanol:acetonitrile:water 5:3:2 *v/v/v*) (Reisz et al., 2018). Samples were vortexed and insoluble material pelleted, as described (Nemkov et al., 2017). Analyses were performed using a Vanquish UHPLC coupled online to a Q Exactive mass spectrometer (Thermo Fisher, Bremen, Germany). Samples were analyzed using a 3 min isocratic condition (Nemkov et al., 2017) or a 5, 9, and 17 min gradient, as described (Nemkov et al., 2019; Reisz et al., 2019). Data analysis was performed through the auxilium of the software MAVEN (Clasquin et al., 2012). Graphs and statistical analyses (either *t*-test or repeated measures ANOVA) were prepared with GraphPad Prism 8.0 (GraphPad Software, Inc., La Jolla, CA, United States), GENE E (Broad Institute, Cambridge, MA, United States), and MetaboAnalyst 5.0 (Pang et al., 2021).

### Hemolysis Measurements

Percent hemolysis was measured based on % hematocrit, supernatant hemoglobin (Hb) (g/dL), and total (Hb) (supernatant + RBC, g/dL) in 50 microliter samples obtained weekly from storage bags. Supernatant and RBCs were separated using a hematocrocrit centrifuge (ThermoFisher, Frederick, MD, United States). Hematocrit was recorded and supernatant was separated from RBCs. Supernatant and lysed RBC Hb levels were measured using a Cary 60 UV-visible spectrophotometer (Agilent Technologies, Santa Clara, CA, United States). Oxy ferrous Hb (HbFe^2+^O_2_) and ferric Hb (HbFe^3+^) concentrations were determined based on the extinction coefficients for each species. Molar extinction coefficients used to calculate Hb concentrations in heme equivalents were: 15.2 mM^–1^ cm^–1^ at 576 nm for Hb(O_2_) and 4.4 mM^–1^ cm^–1^ at 631 nm for ferric Hb using 50 mM potassium phosphate buffer, pH 7.0, at ambient temperature, in both cases. Total heme was calculated by adding these values and converting (heme) (microM) to total (Hb) (g/dL).

### Red Blood Cell Morphological Evaluation

Red blood cells were fixed (1% glutaraldehyde in 0.1 M phosphate buffer) and post-fixed with 1% osmium tetroxide for 1 h at room temperature, prior to further preparation and evaluation by Scanning Electron Microscopy, as described (Stefanoni et al., 2020).

## Results

### Guinea Pig Red Blood Cells Are Distinct Metabolically From Humans, Baboons, and Macaques

**FIGURE 1:**
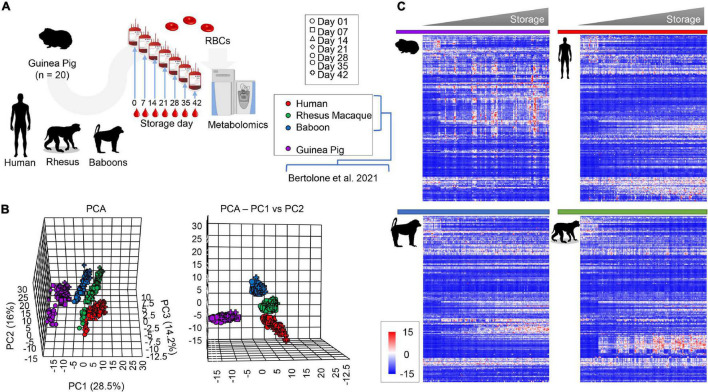
Metabolic phenotypes of stored guinea pig, human, rhesus macaque, and baboon RBCs. Results were determined *via* high-throughput mass spectrometry-based metabolomics **(A)**. Multivariate analyses show distinct metabolic phenotypes at baseline and throughout storage, including principal component analysis [**(B)**, top and front view] and hierarchical clustering analysis of the significant metabolites by two-way ANOVA [**(C)**, time and species].

Metabolomics analyses were performed on leukocyte-filtered packed RBCs from guinea pigs (*n* = 20) at storage day 0, 7, and weekly thereafter until storage day 42; the latter is the FDA mandated shelf-life for human RBC concentrates stored in AS-3 (Figure 1A). Results were compared to metabolomics studies on stored RBCs from humans (*n* = 21), baboons (*n* = 20), and macaques (*n* = 20) RBCs, which we reported previously (Bertolone et al., 2020) (Figure 1A). All the raw data are extensively provided in tabulated form as Supplementary Table 1 and as a heat map in Supplementary Figure 1. Partial least square-discriminant analysis (PLS-DA; Figure 1B) showed a separation between guinea pigs and all primates on principal component 1 (PC1), accounting for 28.5% of the total variance, while PC2 (16%) discriminated across humans and non-human primates (Figure 1B). Storage duration followed a trend from day 0 through 42 along PC3 (14.2%—Figure 1B). A heat map was drawn based on hierarchical clustering analyses of significant metabolites by two-way ANOVA (Figure 1C). These results confirm and expand on previous studies comparing humans and macaques (Stefanoni et al., 2020), and baboons (Bertolone et al., 2020).

### Alteration of Energy and Redox Metabolism in Stored Guinea Pig Red Blood Cells

**FIGURE 2:**
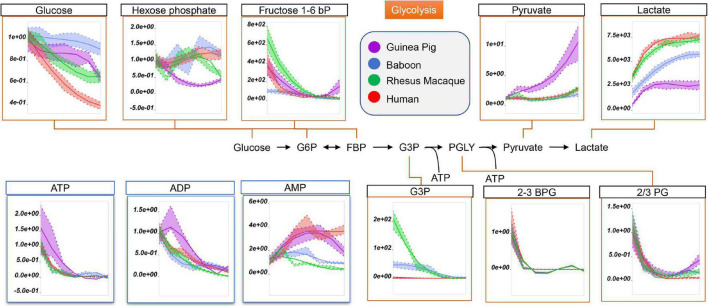
Impact of storage on glycolytic metabolites and high-energy purines (ATP, ADP, AMP) in stored RBCs from guinea pigs (violet), baboons (blue), rhesus macaques (green), and humans (red). Line plots indicate metabolite medians (lighter colored areas are ranges) normalized to measurements in fresh, non-stored blood (day 0) and autoscale normalized across groups. All metabolites are significant by ANOVA (FDR corrected–*p* < 0.05).

Being devoid of organelles, RBCs rely on glucose oxidation *via* the Embden-Meyerhof-Parnas pathway to generate energy in the form of adenosine triphosphate (ATP). Interestingly, RBC levels of ATP, ADP, and AMP in fresh and stored RBCs were the highest in guinea pigs and humans (Figure 2). Refrigerated storage promoted significant glucose consumption in guinea pig RBCs, compared to those of non-human primates, but significantly more slowly than human RBCs (Figure 2). Storage-dependent decreases in all hexose and triose phosphate metabolites, especially 2,3-diphosphoglycerate (2,3-BPG in Figure 2), were observed across all species with no significant interspecies differences (Figure 2). However, guinea pigs showed the lowest levels of hexose phosphate, glyceraldehyde 3-phosphate (G3P), and lactate, but the highest levels of pyruvate (Figure 2).

**FIGURE 3:**
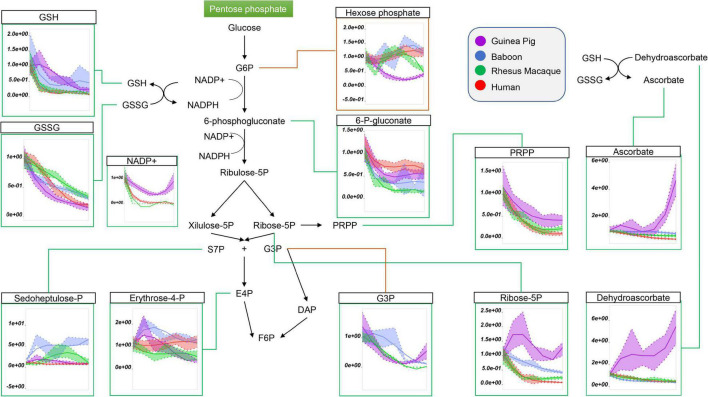
Impact of storage on the pentose phosphate pathway, and on glutathione and ascorbate metabolites in stored RBCs from guinea pigs (violet), baboons (blue), rhesus macaques (green), and humans (red). Line plots indicate metabolite medians (lighter colored areas are ranges) normalized to measurements in fresh, non-stored blood (day 0) and autoscale normalized across groups. All metabolites are significant by ANOVA (FDR corrected–*p* < 0.05).

Because glucose 6-phosphate was the lowest in guinea pig RBCs throughout storage (Figure 2; *nota bene*: hexose phosphate isomers could not be resolved with the analytical high-throughput approach used in this study), we next considered the role of the pentose phosphate pathway (PPP). Steady state levels of oxidative phase PPP metabolites, including 6-phosphogluconate, ribose 5-phosphate (and pentose phosphate isomers), ribose phosphate, and phosphoribosylpyrophosphate were highest in guinea pig RBCs (Figure 3). In contrast, end of storage levels of non-oxidative phase sedoheptulose phosphate and erythrose phosphate were highest in baboon and human RBCs, respectively (Figure 3). In all species, reduced and oxidized glutathione (GSH and GSSG) declined during storage, with the former being the highest, and the latter the lowest, in guinea pig RBCs (Figure 3). Although incapable of *de novo* ascorbate synthesis (Norum and Grav, 2002), guinea pig RBCs had higher total levels of ascorbate and dehydroascorbate at the end of storage, with unexpected increasing trends as a function of storage duration (Figure 3). Altogether, these results are consistent with either activation of antioxidant systems as a function of increased oxidant stress in guinea pig RBCs, or higher basal levels/reservoirs of antioxidant compounds (i.e., vitamin C metabolites), as compared to other species. Indeed, guinea pig RBCs have been previously shown to have an elevated activity superoxide dismutase, catalase and total superoxide scavenger activity in the face of oxidant stress. (Aktan et al., 2003), though to the best of the authors’ knowledge no direct comparative biology study of RBC antioxidant enzymes has been reported to date.

### Increased Purine Oxidation and Dysregulation of Methionine Metabolism in Stored Red Blood Cells From Guinea Pigs, as Compared to Primates

**FIGURE 4:**
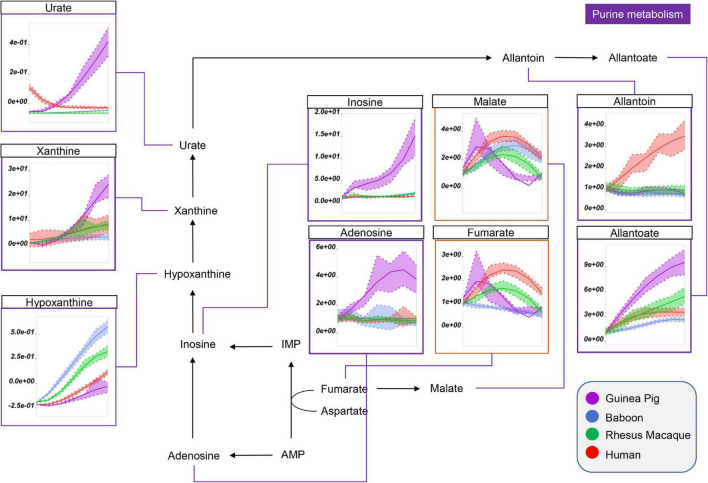
Impact of storage on purine oxidation and carboxylic acid metabolism in RBCs from guinea pigs (violet), baboons (blue), rhesus macaques (green), and humans (red). Line plots indicate metabolite medians (lighter colored areas are ranges) normalized to measurements in fresh, non-stored blood (day 0) and autoscale normalized across groups. All metabolites are significant by ANOVA (FDR corrected–*p* < 0.05).

Interestingly, guinea pig RBCs were also characterized by significantly higher levels of purine metabolites that result from the breakdown of high energy purines (ATP, ADP, AMP) and oxidation [e.g., *via* oxidant stress-triggered RBC-specific AMP deaminase 3 (Nemkov et al., 2018)] (Figure 4). This group includes adenosine, inosine, xanthine, urate (highest in humans at the beginning of storage), and allantoate (Figure 4). Of note, human and guinea pig RBCs had the lowest levels of hypoxanthine, although in humans we observed concomitant increases in purine salvage metabolites (e.g., fumarate; Figure 4), which were lowest in guinea pig and baboon RBCs by the end of storage. Notably, human RBCs also had the highest levels of allantoin, which did not change in the other species throughout storage (Figure 3).

### Altered Amino Acid Metabolism in Guinea Pig Red Blood Cells

**FIGURE 5:**
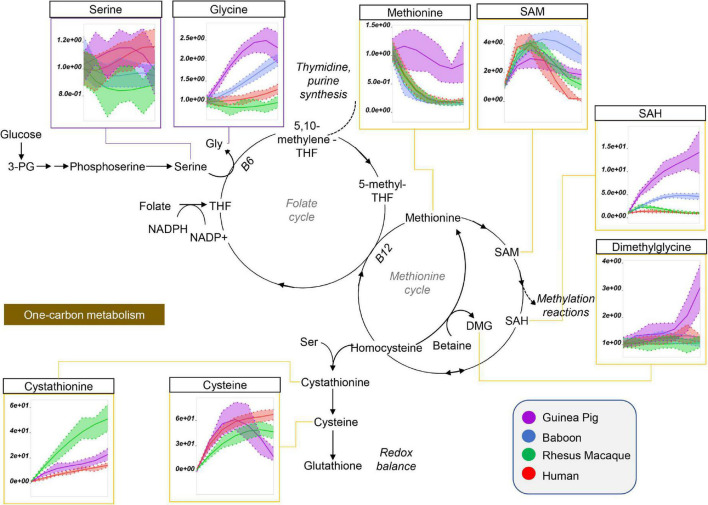
Impact of storage on methionine and one-carbon metabolites in RBCs from guinea pigs (violet), baboons (blue), rhesus macaques (green), and humans (red). Line plots indicate metabolite medians (lighter colored areas are ranges) normalized to measurements in fresh, non-stored blood (day 0) and autoscale normalized across groups. All metabolites are significant by ANOVA (FDR corrected–*p* < 0.05).

**FIGURE 6:**
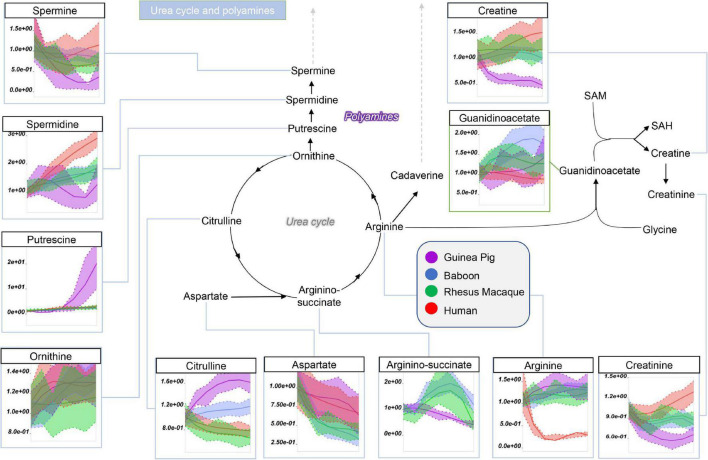
Impact of storage on arginine and polyamine metabolites in RBCs from guinea pigs (violet), baboons (blue), rhesus macaques (green), and humans (red). Line plots indicate metabolite medians (lighter colored areas are ranges) normalized to measurements in fresh, non-stored blood (day 0) and autoscale normalized across groups. All metabolites are significant by ANOVA (FDR corrected–*p* < 0.05).

Guinea pig RBCs had the highest levels of methionine, S-adenosylhomocystine (SAH), glycine, and dimethylglycine and serine, but not of S-Adenosylmethionine (SAM), cysteine, or cystathionine, mostly increasing throughout storage in all species (Figure 5). This is suggestive of dysregulation of one carbon and sulfur metabolism, with up-regulation of methylation events in guinea pig RBCs (lowest SAM/SAH ratios) for handling isoaspartyl-protein damage (Reisz et al., 2018) or purine salvage. Of note, polyamine metabolism was up-regulated at the level of putrescine (highest in guinea pigs), but not spermidine and spermine (lowest in guinea pigs), suggesting storage-dependent activation of ornithine decarboxylase, but not spermidine oxidase, in guinea pig RBCs (Figure 6). Interestingly, citrulline and arginine were highest, and arginine-succinate, guanidinoacetate, creatine, and creatinine lowest, in guinea pig RBCs, suggesting increased urea cycle, but decreased creatine, metabolism (Figure 6). Of note, the effects on arginine metabolism *via* either arginase 1 (producing ornithine) or nitric oxide synthase (producing citrulline) were even more pronounced in guinea pig RBCs than reported the non-human primates (Bertolone et al., 2020), with human RBCs still displaying the lowest arginine levels throughout storage of all tested species.

Relevant to, and consistent with, the data regarding total glutathione pools, guinea pig RBCs had the highest end-of-storage levels of glutathione precursors/catabolites, glutamine, glutamate (and the transamination product 2-oxoglutarate, but not alanine), 5-oxoproline, gamma-glutamyl-cysteine (until storage day 21) (Supplementary Figure 1), and asparagine (full list of amino acids is provided in Supplementary Figure 2). Of note, dysregulation of tryptophan metabolism, with increases in kynurenic acid and indole acetate, was observed in baboons, but not in guinea pigs or other species, with kynurenine increasing during storage only observed in baboons and guinea pigs (Supplementary Figure 3).

### Increased Levels of Free Fatty Acids in Guinea Pig Red Blood Cells Correspond to Increases in Storage Hemolysis and Altered Morphology

**FIGURE 7:**
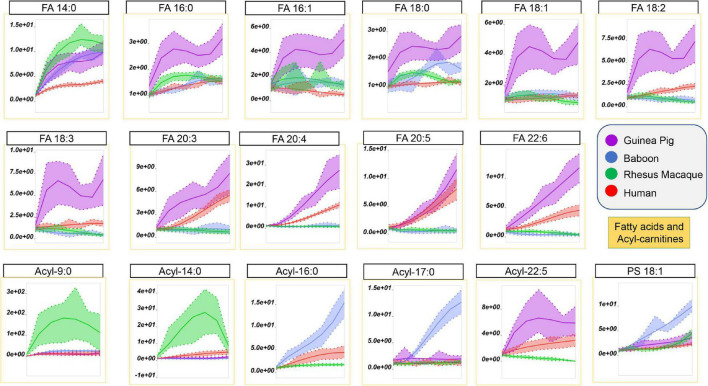
Impact of storage on free fatty acids and acyl-carnitines in RBCs from guinea pigs (violet), baboons (blue), rhesus macaques (green), and humans (red). Line plots indicate metabolite medians (lighter colored areas are ranges) normalized to measurements in fresh, non-stored blood (day 0) and autoscale normalized across groups. All metabolites are significant by ANOVA (FDR corrected–*p* < 0.05).

Storage-dependent increases in the levels of free fatty acids, especially poly- and highly-unsaturated fatty acids, was previously reported for human and non-human primate RBCs (Bertolone et al., 2020; Thomas et al., 2021). However, guinea pig RBCs showed the highest storage-dependent increases in saturated (SFA–14:0, 16:0, 18:1), monounsaturated (MUFA–16:1, 18:1), and poly- or highly-unsaturated fatty acids (PUFA or HUFA–18:2, 18:3, 20:3, 20:4, 20:5, 22:6) fatty acids, as compared to all primate species (Figure 7). Of note, human RBCs did show increases in PUFAs and HUFAs, as compared to SFAs, a unique trait among the four species tested here. The increases in the levels of free fatty acids perhaps results from increased lipolysis by phospholipase enzymes (or phospholipase-like enzymes, such as peroxiredoxin 6) (Fisher, 2018).

**FIGURE 8:**
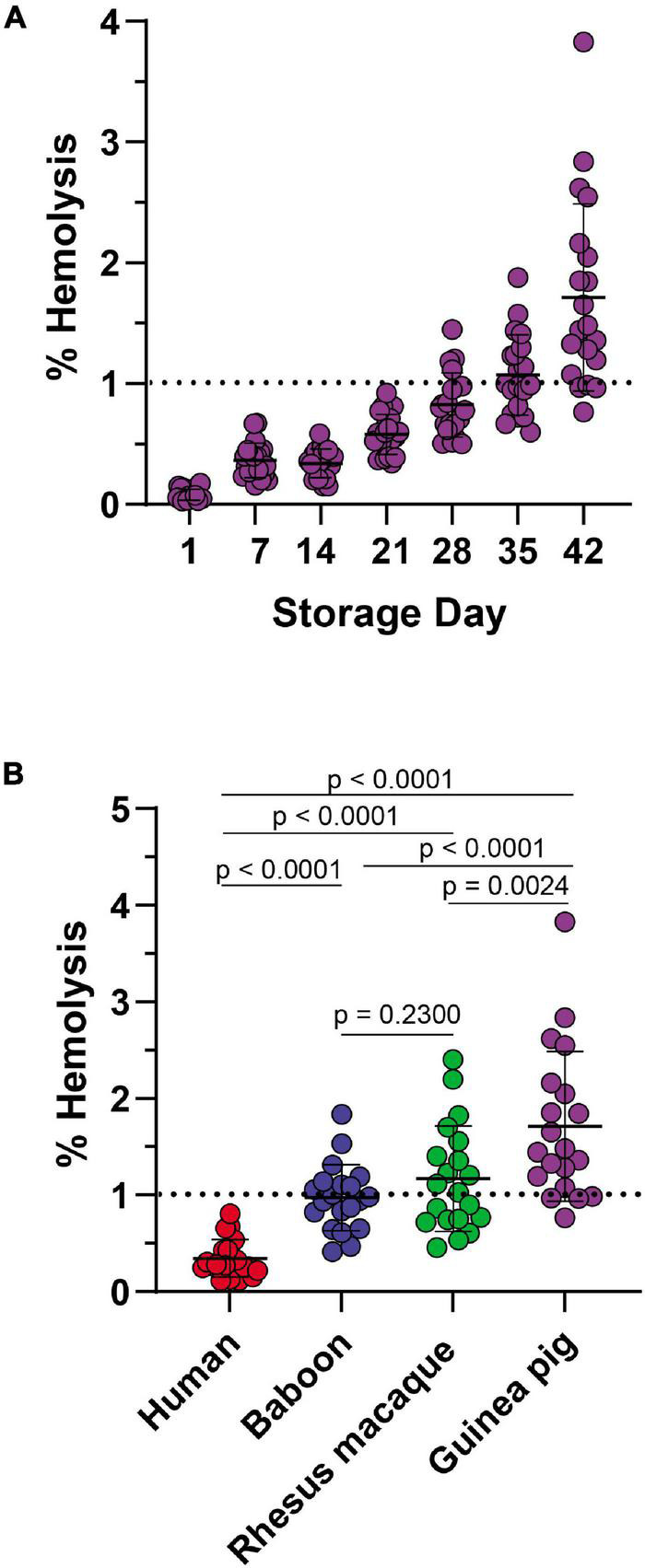
Impact of storage duration on guinea RBC hemolysis **(A)** and comparison to end-of-storage hemolysis in baboons, macaques, and humans **(B)**.

**FIGURE 9:**
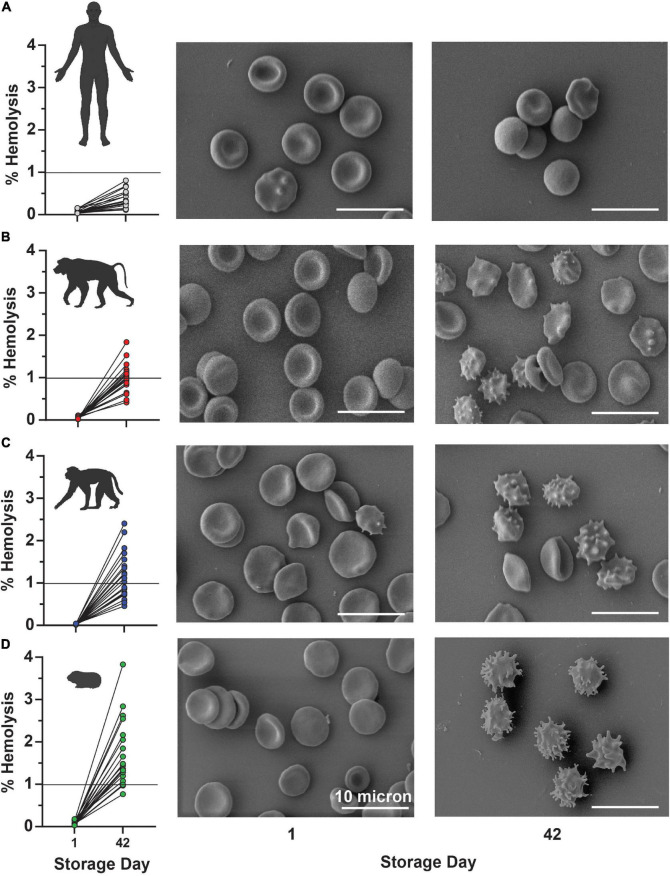
End-of-storage spontaneous hemolysis measurements are directly compared to scanning electron micrographs in fresh (left panels) and 42-day stored (right panel) RBCs from humans **(A)**, baboons **(B)**, macaques **(C)**, and guinea pigs **(D)**.

In keeping with this observation, guinea pig RBCs showed a storage-dependent increase in spontaneous hemolysis (Figure 8A), crossing the 1% Food and Drug Administration mandated threshold for (human) RBC storage quality by day 28 in 4 units and by day 35 in > 50% units. An interspecies comparison of end-of-storage hemolysis confirmed a more significant likelihood of guinea pig RBCs to hemolyze, followed by Rhesus macaques, baboons, and, most protected from spontaneous storage hemolysis, humans (Figure 8B). These results were further validated by Scanning Electron Microscopy, showing the lowest likelihood to develop irreversible morphological alterations (e.g., spheroechinocytes, spherocytes) in humans (Figure 9A), followed by baboons, macaques, and guinea pigs, in the order of increasing damage (Figures 9B–D).

**FIGURE 10:**
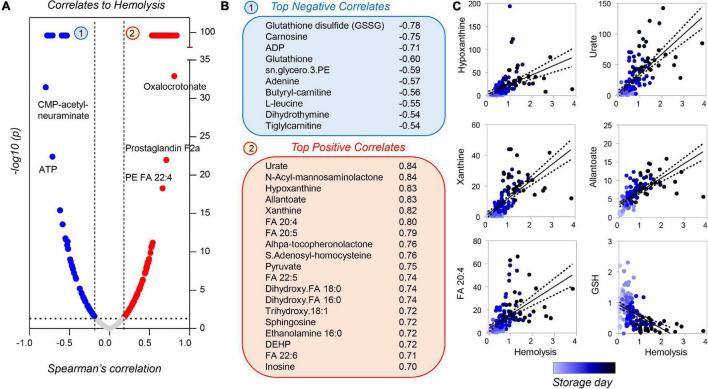
Metabolic correlates to hemolysis for stored guinea pig RBCs. Volcano plots in panel **(A)** indicate Spearman correlation (x-axis) and significance [–log10(*p*-value) on the y-axis]. A subset of metabolites was strongly correlated [arbitrary –log10(p) = 99], either negatively (blue) or positively (red) to storage hemolysis **(B)**. A subset of these metabolites is shown in panel **(C)** (color coded from light to dark blue as a function of storage duration).

Correlation analyses of metabolomics data with storage hemolysis for guinea pig RBCs showed strong positive correlations with purine oxidation and free fatty acids; hemolysis also negatively correlated with glutathione (reduced or oxidized) and ATP levels (Figures 10A–C). These results are consistent with those reported previously in humans and non-human primates (Bertolone et al., 2020; Thomas et al., 2021), with the exception of urate and poly-unsaturated fatty acids, which showed divergent trends in humans as compared to guinea pigs and non-human primates.

## Discussion

Omics approaches to comparative biology of plants (Wei et al., 2002; Izawa et al., 2003) and animals have so far mostly involved genomics. The recent introduction of high-throughput metabolomics has enabled the direct comparison of large sample sets, making it possible not only to perform analyses on hundreds to thousands of samples in a seamless fashion, but also allow for rapid responses to new diseases (D’Alessandro et al., 2021d). Herein we leveraged this technology to investigate the impact of refrigerated storage on guinea pig RBCs and compared these results to previously published data in humans (D’Alessandro et al., 2019), macaques (Stefanoni et al., 2020), and baboons (Bertolone et al., 2020).

The data presented suggest significant storage-induced alterations in hemolysis (Kanias et al., 2017), morphology (Blasi et al., 2012), and energy metabolism (D’Alessandro et al., 2015) in guinea pig RBCs as compared to primates. Specifically, as inferred from steady state measurements, the highest levels of pyruvate and lowest levels of lactate were observed in stored guinea pig RBCs. This observation is consistent with a likely blockade at the lactate dehydrogenase step, a phenomenon previously reported in human RBCs from glucose 6-phosphate dehydrogenase (G6PD)-deficient donors (Tzounakas et al., 2016; Francis et al., 2020; D’Alessandro et al., 2021a). Because lactate dehydrogenase consumes NADH to generate NAD + to keep glycolysis going [i.e., NAD + is required for converting G3P to 1,3-BPG by glyceraldehyde 3-phosphate dehydrogenase (GAPDH)], this result is consistent with alternative routes for NADH recycling in stored guinea pig RBCs. In the face of oxidant stress, methemoglobin reductase [i.e., diaphorase 1 (also termed NADH-cytochrome b5 reductase)] promotes NADH to NAD conversion in a reaction that reduces ferric to ferrous hemoglobin and iron. This pathway is critical to RBC storage lesion severity in that it regulates post-transfusion clearance. In mice this varies due to polymorphisms causing excess activation of the STEAP3 ferrireductase (D’Alessandro et al., 2021c). Similarly, oxidant stress induces fatty acid desaturase-dependent NADH consumption in mature RBCs, a process that is partially observable during storage (Thomas et al., 2021). Of note, storage increased RBC levels of free fatty acids in guinea pig and human RBCs (observed to a lesser extent in macaque and baboon RBCs). However, a different association with hemolysis was observed for PUFAs and HUFAs in guinea pigs, with opposite trends previously reported in humans (Thomas et al., 2021), suggesting less stress to human RBC membrane lipids by the end of storage. These results are consistent with activation of enzymes with phospholipase activity [e.g., peroxiredoxin 6 (Fisher, 2018)], that could exacerbate membrane remodeling in guinea pig RBCs. Nonetheless, these results are not totally unexpected, because rodent RBCs were previously reported to store well for up to 14 days in mice (Hod et al., 2010) and 21 days in rats (Williams et al., 2019). Mice and rats are closer relatives to guinea pigs than primates, although the latter share with guinea pigs the inability to synthesize ascorbate (Burns, 1957).

Guinea pig RBCs demonstrated significant alterations in the levels of methyl-group donors [similar to humans with G6PD deficiency (Ingrosso et al., 2002)]. Guinea pig RBCs were also characterized by higher levels of the byproducts of methylation events (e.g., *S*-adenoyslhomocysteine), a pathway that in RBCs is activated to repair oxidant stress-induced isoaspartyl damage to proteins (D’Alessandro et al., 2021b). Similarly, guinea pig RBCs showed significantly higher levels (and faster accumulation rates) of oxidized purines as a function of storage duration; this pathway depends on oxidant-stress induced breakdown and/or deamination of high-energy purines, which negatively correlates with the capacity of stored human or rodent RBCs to circulate following transfusion (Nemkov et al., 2018). Notably, although urate levels were previously reported to negatively correlate with storage duration and storage hemolysis in humans (Tzounakas et al., 2018; Bertolone et al., 2020), they were positively associated to storage hemolysis in guinea pigs, as previously observed for baboons and macaques (Bertolone et al., 2020; Stefanoni et al., 2020).

Limitations of this study include the focus on steady state analyses, with the lack of flux analysis data. Nonetheless, these results suggest an early (at the fructose bisphosphate to glyceraldehyde 3-phosphate step) and late (pyruvate to lactate) glycolytic blockade, in guinea pig RBCs compared to the other species. They also suggest an exacerbation of oxidant stress-induced oxidation of rate-limiting glyceraldehyde 3-phosphate dehydrogenase, as previously reported with stored human RBCs (Reisz et al., 2016); future (redox) proteomics studies should test this specific hypothesis. Our steady state data are consistent with a bottleneck in glycolysis and redirection of glucose oxidation fluxes toward the PPP to generate reducing equivalents; this is seen more with guinea pig, as opposed to primate, RBCs. This interpretation would also explain increased reduced glutathione levels in guinea pig RBCs, as well as increased ascorbate levels in the face of ablated *de novo* synthesis. Of course, compensatory mechanisms, such as regulation of ascorbate transport, may occur in guinea pig RBCs, as compared to other species. This concept will need to be evaluated in tracing studies with isotope-labeled ascorbate. Alternatively, interspecies divergencies in this pathway may be explained, at least in part, by the evolution of a transport and extracellular reduction system for ascorbate in primates, but not in guinea pigs; this could depend on GLUT1 and Cytochrome B, a membrane-bound cytochrome catalyzing extracellular reduction of Fe^3+^ and ascorbate free radical, the first oxidized form of ascorbic acid (Eigenschink et al., 2021). Direct comparison to other rodents is currently missing, though severe storage hemolysis was reported in mice and rats after 14 (Zimring et al., 2014) and 21 days (Williams et al., 2019), respectively, which would preclude examining the 42 day storage period tested here. On the other hand, despite the lack of animal-specific guidelines on stored blood shelf-life in veterinary units, it is worth noting that it may make little biological sense to compare guinea pig blood to humans for a similar shelf life of 42 days, owing to the ∼30% shorter lifespan of guinea pig RBCs. As such, the present study may represent an exercise in testing whether specific-pathways are indicative of earlier qualitative decay of the guinea pig RBCs. In other rodents (Hay et al., 2021) and in humans (Page et al., 2021), genetic heterogeneity of the blood donor pool was identified as a significant contributor to the biological variability in blood storage quality; although only one guinea pig strain was tested in the current study, this aspect could be evaluated further in the future. The average age of the guinea pigs tested in this study was ∼2.5 months old (young), which could impact the interpretation of the comparison to the other non-human primates at 5 years of age (young) or humans at age 30–75 (young adults to older age adults), owing to the well-established impact of age on the metabolome of stored RBCs (D’Alessandro et al., 2021a).

## Data Availability Statement

The original contributions presented in the study are included in the article/Supplementary Material, further inquiries can be directed to the corresponding author/s.

## Ethics Statement

Human blood was collected under informed consent according to NIH study IRB #99-CC-0168 “Collection and Distribution of Blood Components from Healthy Donors for *in vitro* Research Use” under an NIH-FDA material transfer agreement and in compliance with the Declaration of Helsinki. The patients/participants provided their written informed consent to participate in this study. The animal study was reviewed and approved by FDA White Oak Animal Care and Use protocols 2009-25 (for guinea pigs) and 2018-31 (macaques and baboons).

## Author Contributions

HS, JB, and PB collected and stored the samples. SS, PB, and AD’A provided essential materials and methods to perform the study. PB and YG performed hemolysis and SEM evaluation of RBCs. LB and AD’A performed metabolomics analyses (untargeted and targeted quantitative), tracing experiments, performed data analysis, and prepared figures and tables. AD’A wrote the manuscript. LB, PB, and AD’A modified the first draft of the manuscript. HS, JB, YG, and SS revised the manuscript. All authors contributed to finalizing the manuscript.

## Conflict of Interest

AD’A is a founder of Omix Technologies Inc. AD’A is also a consultant for Altis Biosciences LLC., Rubius Inc., and Forma Inc. AD’A and SS are both consultants for Hemanext Inc. SS is also a consultant for Tioma, Inc., TCIP, Inc., and the Executive Director of the Worldwide Initiative for Rh Disease Eradication (WIRhE). The remaining authors declare that the research was conducted in the absence of any commercial or financial relationships that could be construed as a potential conflict of interest.

## Publisher’s Note

All claims expressed in this article are solely those of the authors and do not necessarily represent those of their affiliated organizations, or those of the publisher, the editors and the reviewers. Any product that may be evaluated in this article, or claim that may be made by its manufacturer, is not guaranteed or endorsed by the publisher.
