# Supplementary figures and images for: ZOOMICS: Comparative Metabolomics of Red Blood Cells From Guinea Pigs, Humans, and Non-human Primates During Refrigerated Storage for Up to 42 Days
Source: Front Physiol. 2022 Mar 21;13:845347. doi: 10.3389/fphys.2022.845347 (PMC8977988; doi:10.3389/fphys.2022.845347)

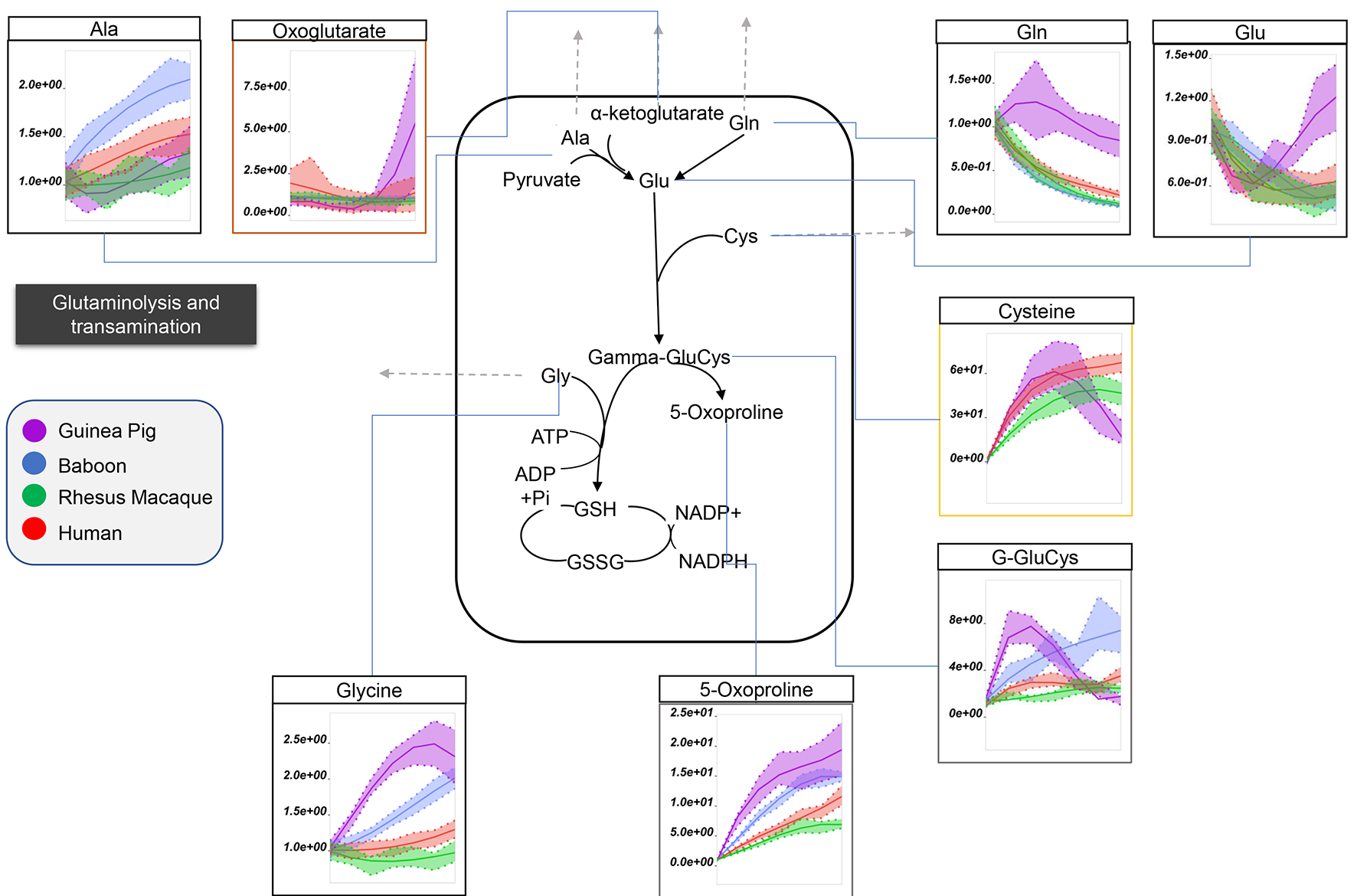

Supplement: Supplementary file 2 [file Image_1.TIF]

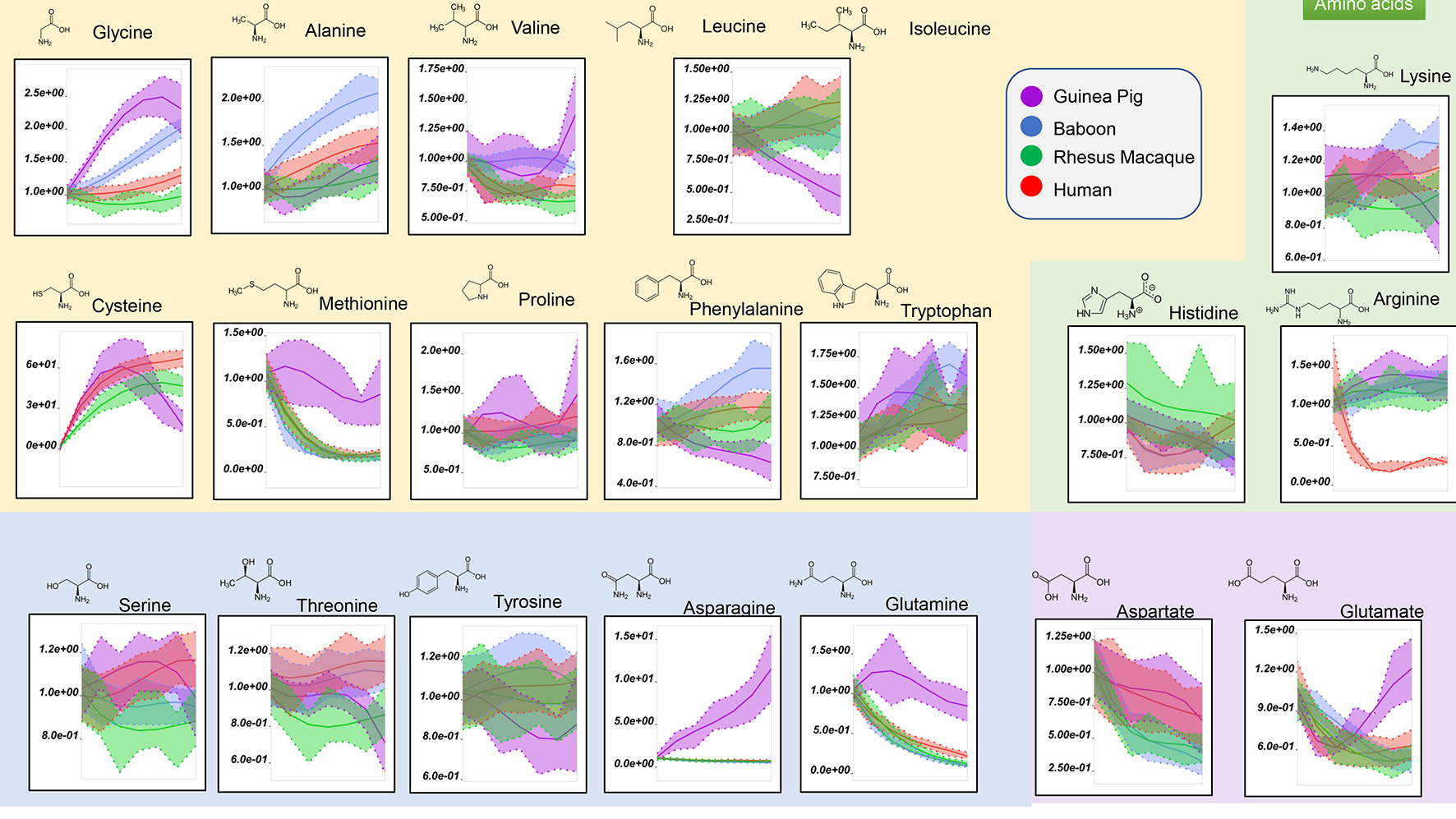

Supplement: Supplementary file 3 [file Image_2.TIF]

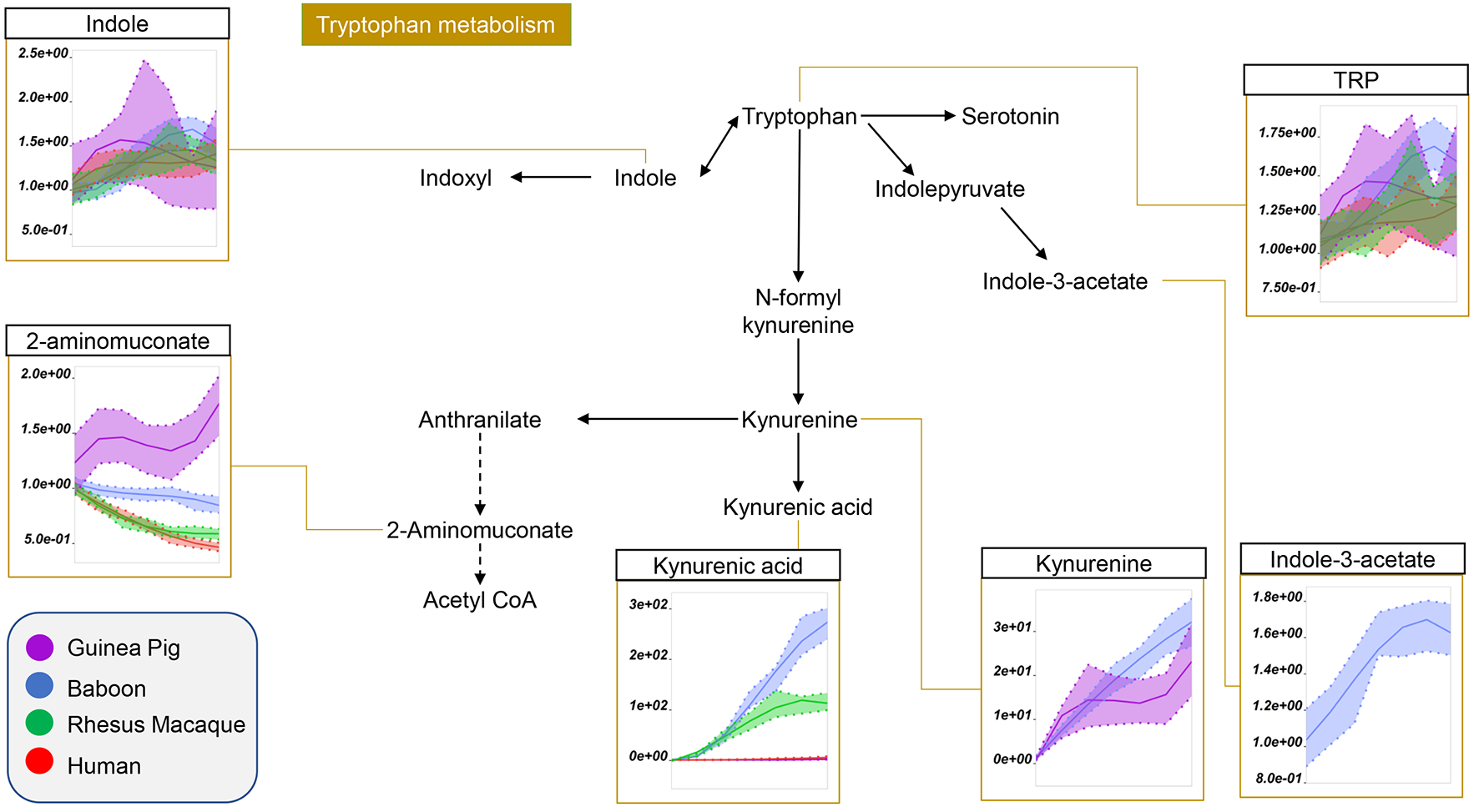

Supplement: Supplementary file 4 [file Image_3.TIF]
